# Data and material of the Safe-Range-Inventory: An assistance tool helping to improve the charging infrastructure for electric vehicles

**DOI:** 10.1016/j.dib.2017.07.061

**Published:** 2017-08-10

**Authors:** Claus-Christian Carbon, Fabian Gebauer

**Affiliations:** University of Bamberg, Department of General Psychology and Methodology, D-96047 Bamberg, Germany

**Keywords:** Electric vehicle, Battery, Charging, Fast-charge, AC/DC, Infrastructure

## Abstract

The Safe-Range-Inventory (SRI) was constructed in order to help public authorities to improve the charging infrastructures for electric vehicles [1; 10.1016/j.trf.2017.04.011]. Specifically, the impact of fast (vs slow) charging stations on people's range anxiety was examined. Ninety-seven electric vehicle users from Germany (81 male; *M*_age_=46.3 years, *SD*=12.1) were recruited to participate in the experimental design. Statistical analyses were conducted using ANOVA for repeated measures to test for interaction effects of available charging stations and remaining range with the dependent variable *range anxiety*. The full data set is publicly available via https://osf.io/bveyw/ (Carbon and Gebauer, 2017) [2].

**Specifications Table**

**Table:** 

Subject area	*Psychology*
More specific subject area	*Traffic Psychology, electromobility usage*
Type of data	*Table, graph, figure*
How data was acquired	*Survey*
Data format	*Raw, analyzed*
Experimental factors	*Multivariate analyses*
Experimental features	*Very brief experimental description*
Data source location	*Germany*
Data accessibility	*Partial data are within the article; the full data set is publicly available via Open Science Framework* (https://osf.io/bveyw/)

**Value of the data**

• The data is important to evaluate the variance of typical Safe-Range-Inventory assessments
• Important to estimate the impact of fast vs. slow charging stations on electric vehicle user's range anxiety
• The data could be used for public authorities to assist in the planning of electric charging infrastructures
• The data is important to conduct recalculations with own analysis tools and methods
• The data could be useful as a starting point for further research on electric users' range anxiety

## 1 Data

This paper contains data of the Safe-Range-Inventory (SRI) based on a recently published paper [1; 10.1016/j.trf.2017.04.011]. It examines how far different charging infrastructure might have an impact on electric vehicle user's range anxiety. The SRI can assist in the planning of electric charging infrastructures in order to find the right balance between range safety and installation and maintenance costs.

## 2 Experimental design, materials and methods

**Fig. 1:**
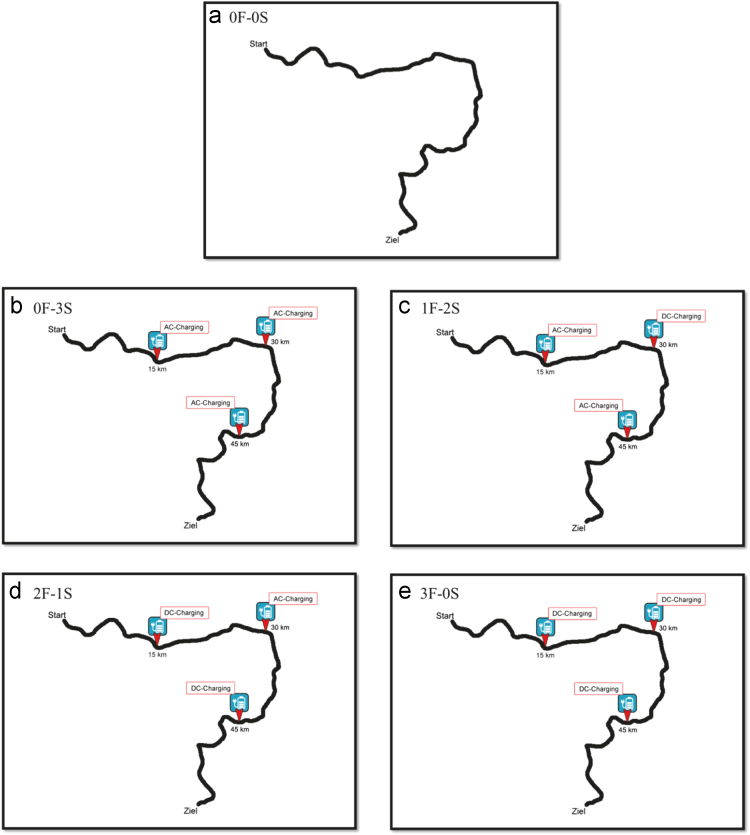
Depiction of the five different scenarios as shown to the participants (German “Ziel” means detination).

**Fig. 2:**
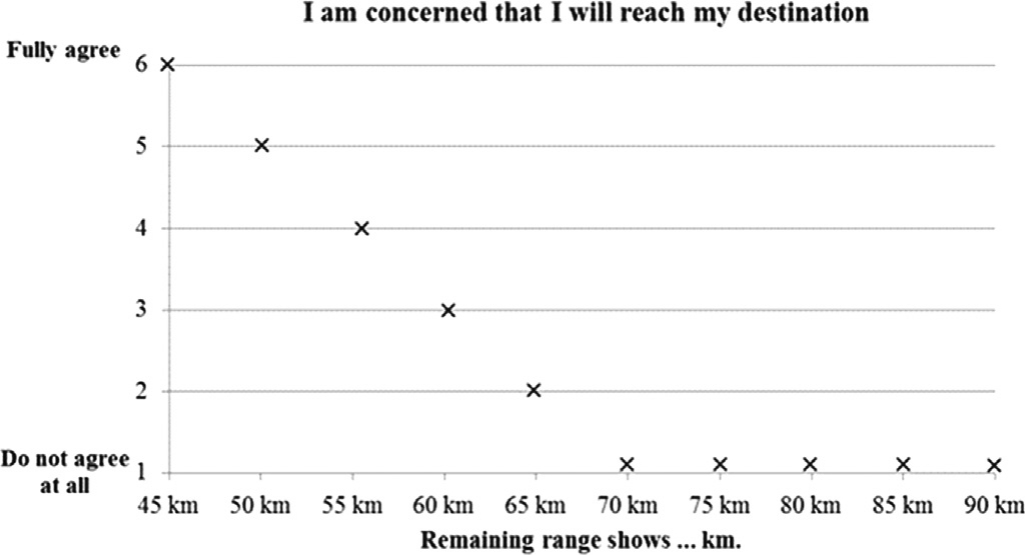
An example of a fictional grid from the Safe-Range-Inventory (SRI) referring to the facet *concerns about reaching the destination*. Participants simply had to tick their assessment for each remaining range (at the start of their trip).

The SRI consists of five scenarios that systematically differed in terms of the number as well as the composition of fast (specific charging times were based on typical 50 kW DC technology) vs. slow (specific charging times were based on typical 4.7 kW AC technology). Each scenario had to be rated on three different facets of range safety/anxiety using a multi-faceted assessment tool based on bi-axial grids. Ninety-seven electric vehicle users from Germany (81 male; *M*_age_=46.3 years, *SD*=12.1) were recruited to participate in the experimental design. Table 1 describes the written material of each scenario while Fig. 1 shows the visualization of the corresponding scenarios being used. Fig. 2 gives an example of a fictional grid from the SRI including the facet *concerns about reaching the destination*.

**Table 1:** Describing the introduction for each scenario.

Standardized introductory part that was the same for all scenarios	Imagine you are in a city and you have an appointment that you want to arrive punctually for. You have to take the route displayed below, which is approximately 60 km long. The traffic is at a daily average level and you need not expect any roadworks or traffic jams. You are driving with your own electric vehicle without a range extender.
Condition a) 0 fast and 0 slow charging stations (0F[fast]-0S[slow])	There are no charging stations available along your route.
Condition b) 0 fast and 3 slow charging stations (0F-3S)	There are 3 slow, conventional charging stations available on your route. Charging at a slow, conventional charging station (with AC technology) takes 6–8 h to recharge an electric vehicle's nearly empty battery up to 80%.
Condition c) 1 fast and 2 slow charging stations (1F-2S)	There are 2 slow, conventional and 1 fast charging stations available on your route. Charging at a slow, conventional charging station (with AC technology) takes 6–8 h to recharge an electric vehicle's nearly empty battery up to 80%. Charging at a fast-charging station (with DC technology) takes 20 min to recharge an electric vehicle's nearly empty battery up to 80%
Condition d) 2 fast and 1 slow charging stations (2F-1S)	There is 1 slow, conventional and 2 fast charging stations available on your route. Charging at a slow, conventional charging station (with AC technology) takes 6–8 h to recharge an electric vehicle's nearly empty battery up to 80%. Charging at a fast-charging station (with DC technology) takes 20 min to recharge an electric vehicle's nearly empty battery up to 80%
Condition d) 3 fast and 0 slow charging stations (3F-0S)	There are 3 fast charging stations available on your route. Charging at a fast-charging station (with DC technology) takes 20 min to recharge an electric vehicle's nearly empty battery up to 80%

For each facet we calculated an ANOVA for repeated measures using the within-subject factors scenario × remaining range (Table 2) and additionally applied Bonferroni corrected pairwise comparisons (Table 3).

**Table 2:** Statistical analyses concerning the main effects *condition*, *remaining range* and their interaction effects.

Facet	Main effect *condition*			Main effect *remaining range*			Interaction effect *condition* × *remaining range*		
	*F*	*p*	η_p_^2^	*F*	*p*	η_p_^2^	*F*	*p*	η_p_^2^
1^st^ facet: *I am concerned whether I will reach my destination.*	74.79	<.001	.76	22.90	<.001	.70	16.35	<.001	.90
2^nd^ facet: *I am not worried about my EV's range along this route.*	52.29	<.001	.69	22.65	<.001	.70	13.06	<.001	.89
3^rd^ facet: *I am sure that I will reach my destination with my EV on time.*	36.83	<.001	.62	37.46	<.001	.80	7.80	<.001	.83

**Table 3:** Showing descriptive data of participant's ratings for every fact and every condition (*N*=97).

	Condition	Remaining range (in km)																			
		45		50		55		60		65		70		75		80		85		90	
		*M*	*SD*	*M*	*SD*	*M*	*SD*	*M*	*SD*	*M*	*SD*	*M*	*SD*	*M*	*SD*	*M*	*SD*	*M*	*SD*	*M*	*SD*
Facet 1: "Concerns"	0F-0S	5.56	1.22	5.27	1.42	4.72	1.68	3.83	1.92	3.05	1.83	2.33	1.61	1.76	1.32	1.45	1.07	1.25	0.94	1.16	0.78
	0F-3S	3.04	2.24	2.73	2.13	2.33	1.95	2.01	1.78	1.74	1.52	1.46	1.18	1.30	0.95	1.13	0.64	1.13	0.63	1.08	0.51
	1F-2S	2.17	1.89	1.97	1.75	1.82	1.63	1.63	1.52	1.38	1.19	1.25	0.94	1.21	0.84	1.17	0.77	1.13	0.69	1.17	0.80
	2F-1S	2.11	1.89	1.87	1.69	1.76	1.58	1.56	1.43	1.31	1.06	1.20	0.86	1.17	0.80	1.16	0.77	1.13	0.74	1.11	0.68
	3F-0S	1.63	1.46	1.46	1.23	1.29	1.01	1.16	0.63	1.10	0.57	1.07	0.53	1.07	0.53	1.08	0.52	1.04	0.25	1.06	0.43
																					
Facet 2: "Not worried"	0F-0S	1.44	1.30	1.70	1.44	2.32	1.78	3.18	2.02	3.88	1.90	4.71	1.65	5.29	1.29	5.53	1.10	5.72	0.93	5.83	0.79
	0F-3S	3.83	2.17	4.03	2.14	4.44	2.03	4.76	1.89	5.14	1.59	5.43	1.31	5.67	1.03	5.75	0.90	5.80	0.80	5.86	0.71
	1F-2S	4.85	1.85	5.00	1.74	5.09	1.70	5.30	1.60	5.63	1.13	5.71	0.99	5.77	0.80	5.83	0.69	5.89	0.52	5.93	0.41
	2F-1S	4.83	1.92	4.97	1.80	5.12	1.69	5.27	1.57	5.54	1.28	5.66	1.12	5.74	0.98	5.77	0.93	5.80	0.85	5.85	0.78
	3F-0S	5.02	1.81	5.25	1.58	5.40	1.45	5.59	1.23	5.67	1.12	5.79	0.92	5.81	0.89	5.86	0.82	5.89	0.66	5.94	0.52
																					
Facet 3: "On time"	0F-0S	1.35	1.08	1.48	1.22	2.08	1.71	3.00	2.02	3.85	1.91	4.68	1.65	5.25	1.30	5.61	1.02	5.73	0.92	5.85	0.74
	0F-3S	2.60	2.01	2.90	2.03	3.50	2.04	4.16	2.08	4.73	1.83	5.20	1.54	5.59	1.13	5.70	0.95	5.77	0.84	5.83	0.75
	1F-2S	3.80	2.10	4.04	2.02	4.41	1.93	4.83	1.79	5.40	1.26	5.61	1.08	5.76	0.77	5.87	0.51	5.91	0.38	5.95	0.27
	2F-1S	3.87	2.12	4.07	2.04	4.42	1.98	4.85	1.84	5.27	1.53	5.72	0.95	5.75	0.82	5.80	0.72	5.89	0.50	5.94	0.38
	3F-0S	4.00	2.03	4.21	1.95	4.58	1.89	5.01	1.66	5.34	1.38	5.72	0.90	5.80	0.89	5.84	0.76	5.87	0.69	5.80	0.59

Every condition was accompanied by a map visualizing the route and the points where charging stations were available (Fig. 1) just to illustrate the different scenarios in order to support the participants’ mental images of them.

After having elaborated upon each scenario, the participants were asked to express their assessment on different facets of range safety/anxiety by means of the so-called *Safe-Range-Inventory*, which we have constructed as a multi-faceted assessment tool based on bi-axial grids. The x-axis of these grids always presents the electric vehicle's remaining range at the start of the trip (for an example see Fig. 2). The meaning of the y-axes across the inventory's items was changed to capture range safety/anxiety in a multi-faceted way (1^st^ facet: *I am concerned whether I will reach my destination*; 2^nd^ facet: *I am not worried about my EV's range along this route*, 3^rd^ facet: *I am sure that I will reach my destination with my EV on time*.). We chose these facets in order to measure general concerns with the EV's range (1^st^ facet), to have valid data on a reversed item (2^nd^ facet) and to measure whether fast and slow-charging stations might affect participant's concerns about punctuality (3^rd^ facet). For usability reasons, we always utilized the same 6-points Likert scale for the y-axis where the end points were operationalized as “1*=do not agree at all*” and “6=*fully agree*”. The grid structure (see Fig. 2) allows for an economic and usable assessment as each grid actually represents a number of items, in the given case 10 single items regarding assessments for the remaining ranges between 45 km and 90 km in steps of 5 km.

In Fig. 2 we will show descriptive analysis of the data for facet *concerns about reaching the destination*. For each facet we calculated an ANOVA for repeated measures (Table 2) using the within-subject factors condition (0F-0S; 0F-3S; 1F-2S; 2F-1S; 3F-0S)×remaining range (45–90 km; in steps of 5 km). Additionally, we depicted means and standard deviation for every facet and every condition (Table 3).
