# Supplementary material for: Data and material of the Safe-Range-Inventory: An assistance tool helping to improve the charging infrastructure for electric vehicles
Source: Data Brief. 2017 Aug 10;14:573–8. doi: 10.1016/j.dib.2017.07.061 (PMC5570577; doi:10.1016/j.dib.2017.07.061)
Supplement: Supplementary file 1 — Transparency document [file mmc1.docx]

*Data article*

**Title:** Data and material of the Safe-Range-Inventory: An assistance tool helping to improve the charging infrastructure for electric vehicles

**Authors:** Claus-Christian Carbon & Fabian Gebauer

**Affiliations:** University of Bamberg, Department of General Psychology and Methodology, D-96047 Bamberg, Germany

**Contact email:** ccc@experimental-psychology.com

**Keywords:** electric vehicle; battery; charging; fast-charge; AC/DC; infrastructure

**Conflicts of interest: none**
